# Higher incidence of perivalvular abscess determines perioperative clinical outcome in patients undergoing surgery for prosthetic valve endocarditis

**DOI:** 10.1186/s12872-020-01338-y

**Published:** 2020-02-03

**Authors:** Carolyn Weber, Parwis B. Rahmanian, Melanie Nitsche, Asmae Gassa, Kaveh Eghbalzadeh, Stefanie Hamacher, Julia Merkle, Antje-Christin Deppe, Anton Sabashnikov, Elmar W. Kuhn, Oliver J. Liakopoulos, Thorsten Wahlers

**Affiliations:** 1 Department of Cardiothoracic Surgery, University of Cologne, Kerpener Strasse 62, D-50937 Cologne, Germany; 2 Institute of Medical Statistics and Computational Biology, Faculty of Medicine and University Hospital of Cologne, University of Cologne, Cologne, Germany

**Keywords:** Prosthetic valve endocarditis, PVE, Perivalvular infection, Perivalvular abscess

## Abstract

**Background:**

Cardiac surgery for prosthetic valve endocarditis (PVE) is associated with substantial mortality. We aimed to analyze 30-day and 1-year outcome in patients undergoing surgery for PVE and sought to identify preoperative risk factors for mortality with special regard to perivalvular infection.

**Methods:**

We retrospectively analyzed data of 418 patients undergoing valve surgery for infective endocarditis between January 2009 and July 2018. After 1:1 propensity matching 158 patients (79 PVE/79 NVE) were analyzed with regard to postoperative 30-day and 1-year outcomes. Univariate and multivariable analyses were performed to identify potential risk factors for mortality.

**Results:**

315 patients (75.4%) underwent surgery for NVE and 103 (24.6%) for PVE. After propensity matching groups were comparable with regard to preoperative characteristics, clinical presentation and microbiological findings, except a higher incidence of perivalvular infection in patients with PVE (51.9%) compared to NVE (26.6%) (*p* = 0.001), longer cardiopulmonary bypass (166 [76–130] vs. 97 [71–125] min; *p* < 0.001) and crossclamp time (95 [71–125] vs. 68 [55–85] min; p < 0.001). Matched patients with PVE showed a 4-fold increased 30-day mortality (20.3%) in comparison with NVE patients (5.1%) (*p* = 0.004) and 2-fold increased 1-year mortality (PVE 29.1% vs. NVE 13.9%; *p* = 0.020). Multivariable analysis revealed perivalvular abscess, sepsis, preoperative AKI and PVE as independent risk factors for mortality. Patients with perivalvular abscess had a significantly higher 30-day mortality (17.7%) compared to patients without perivalvular abscess (8.0%) (*p* = 0.003) and a higher rate of perioperative complications (need for postoperative pacemaker implantation, postoperative cerebrovascular events, postoperative AKI). However, perivalvular abscess did not influence 1-year mortality (20.9% vs. 22.3%; *p* = 0.806), or long-term complications such as readmission rate or relapse of IE.

**Conclusions:**

Patients undergoing surgery for PVE had a significantly higher 30-day and 1-year mortality compared to NVE. After propensity-matching 30-day mortality was still 4-fold increased in PVE compared to NVE.

Patients with perivalvular abscess showed a significantly higher 30-day mortality and perioperative complications, whereas perivalvular abscess seems to have no relevant impact on 1-year mortality, the rate of readmission or relapse of IE.

## Background

Among patients undergoing valve operation for infective endocarditis (IE), surgery for prosthetic valve endocarditis (PVE) has been associated with distinct mortality rates in cardiothoracic surgery [1–3]. PVE accounts for 10–30% of all cases of IE and occurs in 1–6% of patients with valve prostheses [4–6]. Clinical presentation is often atypical and negative echocardiographic findings are more common, leading to lower sensitivity of the Duke criteria in patients with suspected PVE [7]. Hence, timely diagnosis of endocarditis is more difficult in presence of a prosthetic valve compared with a native valve [5, 8, 9]. One of the reasons, for the higher rate of negative echocardiographic findings in PVE is, that vegetations occur less frequent, whereas periannular extensions are more common, which are more difficult to detect in echocardiography [4, 5].

Several factors have been associated with bad prognosis in PVE. Perivalvular infection is of a greater concern in PVE than NVE and occurs in 56–100% of PVE patients [1, 4, 10]. Previous studies suggested, that perivalvular infection worsens the prognosis, the perioperative mortality and the risk for reinfection or relapse of endocarditis [11, 12]. The extension of the infection beyond the valve annulus leads to technically more demanding operations, requiring radical debridement and reconstruction [10].

Therefore, we aimed to compare 30-day and 1-year outcome after valve surgery for NVE and PVE. Our second objective was to identify potential preoperative risk factors for mortality with special regard to perivalvular infection.

## Methods

### Study design

We performed a retrospective single-center analysis. Relevant clinical data of all consecutive patients undergoing surgery for IE between January 2009 and July 2018 were extracted from our institutional database.

### Definition of IE and indication for surgery

IE was defined according to the recent modified Duke Criteria [13]. Surgery for IE was indicated according to the recent ESC guidelines for the management of infective endocarditis [9] and performed as previously described [14].

### Data collection

Patients’ demographics, predisposing risk factors and symptoms at the time of onset of IE, echocardiographic and microbiological findings, perioperative data and relevant clinical outcomes were recorded. IE relevant 30-day and 1-year outcomes were reported for hospital stay and at follow-up, respectively. Follow-up was obtained by review of hospital medical records and interview of the patient’s physician. Median duration of the follow-up was 2.05 years [interquartile range (IQR) 0.04–4.69] with a completeness of 76.3%. The follow-up time for survival was measured from the date of operation to either the date of death or the date of the last contact with the patient. The study protocol was approved by the institutional review board (Ethics Committee of the Medical Faculty, University of Cologne, 17–407). Individual informed consent was waived due to the retrospective nature of the collected data.

### Reporting mortality

30-day mortality (day 1–30) was reported as all-cause mortality within the first 30 days after surgery for IE, regardless of the patient’s location (at home or health care facility). 1-year mortality was reported as all-cause mortality occurring between day 31 and 365 after initial surgery. Long-term mortality was defined as all-cause mortality after day 365 and Kaplan-Meier analysis was used to test for differences in long-term survival .

### Statistical analysis

Patients’ characteristics and pre-operative factors were described using mean values ± standard deviation (SD), median [(IQR)], or frequencies and percentages as indicated. Depending on data distribution group differences were compared using unpaired t-test, Mann-Whitney U test, Chi-squared test or Fisher’s exact test as appropriate. Log-rank test was used to test for differences in long-term mortality between PVE and NVE. A 1:1 propensity score matching was performed to exclude potential confounders between groups with a 0.01 caliper width. This propensity score-based matching procedure resulted in a total number of 158 patients. Matching variables were the following preoperative characteristics that showed statistically significant differences or that were considered clinically significant based on previous research: age, sex, aortic/mitral valve IE, preoperative acute kidney injury (AKI) and *Staphylococcus aureus* as causative microorganism*.* Potential risk factors for 30-day mortality (day 1–30) were assessed using logistic regression. We decided not to include EuroSCORE into the univariate analysis because EuroSCORE is per definition higher in patients with previous cardiac surgery. After univariate analysis all variables with a *p*-value less than 0.1 (female gender, age > 65 years, PVE, preoperative AKI, preoperative sepsis, perivalvular abscess, IE with *Staphylococcus aureus*) were entered into the multivariable model using a forward selection (likelihood ratio, p_in_ = 0.05). Results are presented as odds ratio (OR) for 30-day mortality with corresponding 95% confidence interval (CI) and *p*-value. All reported *p*-values are two-sided and considered statistically significant if ≤5%. Statistical analyses were performed using SPSS Statistics Version 25 (IBM Corp., Armonk, NY, USA).

## Results

### Preoperative characteristics and risk factors

**Fig. 1:**
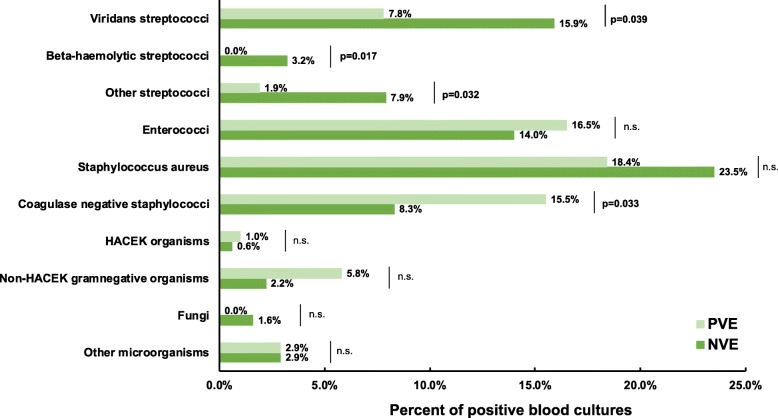
Distribution of causative microorganisms in patients undergoing surgery for native (NVE) versus prosthetic valve endocarditis (PVE)

Data of 418 patients undergoing surgery for infective endocarditis were retrospectively analyzed. 315 patients (75.4%) underwent surgery for NVE and 103 patients (24.6%) for PVE. Table 1 summarizes patients’ demographics and preoperative characteristics. PVE patients in the unmatched cohort were significantly older (71.5 [62.0–76.6] vs. 62.7 [49.4–71.6]; *p* < 0.001), showed a higher proportion of female patients being affected (32.0% vs. 22.5%; *p* = 0.046) and were diagnosed with more preoperative AKI (67.0% vs. 54.9%; *p* = 0.031). In addition, the manifestation of IE differed between PVE and NVE patients (Table 2). The involvement of the aortic valve was more common in the PVE cohort (78.6% vs. 52.7%, *p* < 0.001), whereas mitral valve involvement occurred more often in NVE patients (54.3% vs. 25.2%, p < 0.001). Echocardiography revealed vegetations in 82.2% of NVE and 70.9% of PVE patients (*p* = 0.017). Inversely, PVE patients were diagnosed with more perivalvular infection. Hence, perivalvular abscess was diagnosed in 60.2% of the PVE and 27.0% of the NVE group (*p* < 0.001). Concerning the underlying microorganisms, *Coagulase-negative Staphylococci (CoNS)* were detected more often in PVE (15.5% vs. 8.3%; *p* = 0.033), whereas *Streptococcus spp* were more prevalent in NVE (26.7% vs. 9.7%; *p* < 0.001). The proportion of *Staphylococcus aureus* IE was comparable and occurred in PVE with 18.4% and NVE with 23.5% (*p* = 0.285)*.* Clinical symptoms were similar among both groups (Table 2). Detailed findings regarding causative microorganisms are depicted in Fig. 1.

**Table 1:** Patients’ demographics and preoperative characteristics

	ENTIRE COHORT					PROPENSITY MATCHED COHORT				
	NVE (n = 315)		PVE (n = 103)		P value	NVE (n = 79)		PVE (n = 79)		P value
Age	62.7	[49.4–71.6]	71.5	[62.0–76.6]	< 0.001	65.5	[55.5–72.2]	69.2	[55.5–75.5]	0.204
Female sex	71	(22.5%)	33	(32.0%)	0.046	22	(27.8%)	19	(24.1%)	0.586
BMI	25.5	[23.2–28.1]	26.0	[23.9–28.7]	0.273	25.4	[23.7–28.8]	26.1	[23.9–28.7]	0.548
BSA	1.98	[1.83–2.12]	1.94	[1.74–2.10]	0.238	1.97	± 0.21	1.97	± 0.24	0.349
COPD	29	(9.2%)	9	(8.7%)	0.886	10	(12.7%)	8	(10.1%)	0.617
Diabetes	81	(25.7%)	34	(33.0%)	0.150	21	(26.6%)	25	(31.6%)	0.484
Peripheral vascular disease	23	(7.3%)	12	(11.7%)	0.167	6	(7.6%)	7	(8.9%)	0.772
Preoperative AKI	173	(54.9%)	69	(67.0%)	0.031	54	(68.4%)	52	(65.8%)	0.735
Preoperative dialysis	30	(9.5%)	13	(12.6%)	0.889	6	(7.6%)	13	(16.5%)	0.176
Coronary artery disease	80	(25.4%)	36	(35.5%)	0.060	23	(29.1%)	22	(27.8%)	0.860
Prior PCI	18	(5.7%)	14	(13.6%)	0.054	4	(5.1%)	8	(10.1%)	0.124
Immunosuppression	5	(1.6%)	2	(1.9%)	0.811	1	(1.3%)	1	(1.3%)	1.000
HIV	9	(2.9%)	1	(1.0%)	0.233	0	(0%)	1	(1.3%)	0.238
Alcohol abuse	37	(11.7%)	4	(3.9%)	0.020	8	(10.1%)	3	(3.8%)	0.118
Intravenous drug abuse	22	(7.0%)	6	(5.8%)	0.683	5	(6.3%)	6	(7.6%)	0.755
History of neoplasm	33	(10.5%)	10	(9.7%)	0.824	12	(15.2%)	6	(7.6%)	0.133
LVEF										
< 30%	7	(2.2%)	2	(1.9%)	0.865	1	(1.3%)	2	(2.5%)	0.559
30–50%	63	(20%)	30	(29.1%)	0.532	16	(20.3%)	23	(29.1%)	0.268
> 50%	238	(75.6%)	70	(70.0%)	0.129	62	(78.5%)	53	(67.1%)	0.108
NYHA class										
I + II	55	(17.5%)	19	(18.4%)	0.925	20	(25.3%)	14	(17.7%)	0.245
III + IV	251	(80.0%)	80	(77.7%)	0.662	59	(74.7%)	63	(79.7%)	0.448
Log. EuroSCORE	7.6	[4.4–18.1]	9.8	[22.3–36.2]	< 0.001	7.9	[4.1–16.3]	17.5	[7.9–33.7]	< 0.001
EuroSCORE II	7.0	[5.0–10.0]	11.0	[8.0–13.0]	< 0.001	7.0	[5.0–9.0]	10.0	[7.0–12.3]	< 0.001

Data presented as mean ± standard deviation, number (percent) or median [IQR], respectively. *AKI,* acute kidney injury; *BMI,* body mass index; *BSA*, body surface area; *COPD,* chronic obstructive pulmonary disease; *HIV*, human immunodeficiency virus; *IQR*, interquartile range; *NYHA,* New York Heart Association; *PCI,* percutaneous coronary intervention; *TIA,* transient ischemic attack

**Table 2:** Manifestation of IE according to the modified Duke Criteria

	ENTIRE COHORT					PROPENSITY MATCHED COHORT				
	NVE (n = 315)		PVE (n = 103)		P value	NVE (n = 315)		PVE (n = 103)		P value
MICROBIOLOGY										
Positive Blood culture	263	(83.5%)	77	(74.8%)	0.279	65	(82.3%)	58	(73.4%)	0.505
*Streptococcus spp*	84	(26.7%)	10	(9.7%)	< 0.001	20	(25.3%)	8	(10.1%)	0.011
*Staphylococcus spp*	98	(31.1%)	35	(34.0%)	0.587	20	(25.3%)	24	(30.4%)	0.478
*Staph. aureus*	74	(23.5%)	19	(18.4%)	0.285	16	(20.3%)	15	(19.0%)	0.841
*CoNS*	26	(8.3%)	16	(15.5%)	0.033	5	(6.3%)	9	(11.4%)	0.263
*Enterococcus spp*	44	(14.0%)	17	(16.5%)	0.527	15	(19.0%)	16	(20.3%)	0.841
ECHOCARDIOGRAPHY										
Vegetation	259	(82.2%)	73	(70.9%)	0.017	65	(82.3%)	59	(74.7%)	0.307
Vegetation length (cm)	1.6	[1.1–2.0]	1.2	[0.8–1.7]	0.009	1.5	± 0.6	1.3	± 0.7	0.357
Leftsided IE										
*Aortic valve*	166	(52.7%)	81	(78.6%)	< 0.001	52	(65.8%)	58	(73.4%)	0.299
*Mitral valve*	171	(54.3%)	26	(25.2%)	< 0.001	23	(29.1%)	26	(32.9%)	0.606
Rightsided IE										
*Tricuspid valve*	21	(6.7%)	1	(1.0%)	0.046	6	(7.6%)	1	(1.3%)	0.053
*Pulmonary valve*	1	(0.3%)	1	(1.0%)	0.439	0	(0%)	1	(1.3%)	0.238
Perivalvular infection										
*Perivalvular abscess*	85	(27.0%)	62	(60.2%)	< 0.001	21	(26.6%)	41	(51.9%)	0.001
*Perforation*	83	(26.3%)	21	(20.4%)	0.224	10	(12.7%)	19	(24.1%)	0.064
*Fistula*	1	(0.3%)	13	(12.6%)	< 0.001	1	(1.3%)	10	(12.7%)	0.005
SYMPTOMS										
Fever	201	(63.8%)	72	(69.9%)	0.259	60	(75.9%)	55	(69.6%)	0.371
Sepsis	233	(74.0%)	68	(66.0%)	0.119	41	(51.9%)	38	(48.1%)	0.633
IE-related neurologic complications	99	(31.4%)	28	(27.2%)	0.416	23	(29.1%)	22	(27.8%)	0.860
*TIA*	13	(4.1%)	1	(1.0%)	0.122	3	(3.8%)	0	(0%)	0.305
*Stroke*	47	(14.9%)	17	(16.5%)	0.698	11	(13.9%)	14	(17.7%)	0.513
*Intracranial bleeding*	5	(1.6%)	3	(2.9%)	0.394	1	(1.3%)	3	(3.8%)	0.311
*Other*	33	(10.5%)	7	(6.8%)	0.270	8	(10.1%)	5	(6.3%)	0.385
Septic embolism	115	(36.5%)	29	(28.2%)	0.188	25	(31.6%)	24	(30.4%)	0.239
Cardiogenic shock	46	(14.6%)	9	(8.7%)	0.126	7	(8.9%)	8	(10.1%)	0.786

Data presented as mean ± standard deviation, number (percent) or median [IQR], respectively. *d,* days*; IE* infective endocarditis; *IQR,* interquartile range; *TIA,* transient ischemic attack

After propensity matching there were no statistically significant differences regarding preoperative characteristics, clinical symptoms and manifestation of IE, except a higher rate of perivalvular infection (51.9% vs. 26.6%, *p* = 0.001) (Tables 1 and 2). Due to the complexity of operation, CPB and crossclamp time were significantly longer in patients with PVE (CPB time: 166 [76–130] vs. 97 [71–125] min; *p* < 0.001; crossclamp time: 95 [71–125] vs. 68 [55–85] min; p < 0.001) (Additional file 1: Table S1).

### 30-day and 1-year clinical outcomes

30-day and 1-year clinical outcomes are depicted in Table 3. With regard to 30-day outcome, PVE was associated with a significantly higher 30-day mortality. After propensity-matching 30-day mortality was still 4-fold increased in PVE compared to NVE (20.3% vs. 5.1%; *p* = 0.004). Concerning the incidence of postoperative complications, matched cohorts were comparable except a longer time of ventilation (28.3 [15–113] vs. 16.8 [12–46] hours; *p* = 0.017), longer ICU (5.0 [2.0–12.0] vs. 3.0 [1.0–6.0] days; *p* = 0.042) and hospital stay (15.0 [11.0–20.0] vs. 12.0 [7.0–17.0] days; *p* = 0.015) in the PVE cohort.

**Table 3:** 30-day and 1-year clinical outcomes

	ENTIRE COHORT					PROPENSITY MATCHED COHORT				
	NVE (n = 315)		PVE (n = 103)		P value	NVE (n = 315)		PVE (n = 103)		P value
30-DAY OUTCOME										
30-day mortality	26	(8.3%)	22	(21.4%)	**< 0.001**	4	(5.1%)	16	(20.3%)	**0.004**
Myocardial infarction	1	(0.3%)	1	(1.0%)	0.439	0	(0%)	1	(1.3%)	0.238
New pacemaker *	23	(7.3%)	20	(19.4%)	**< 0.001**	8	(10.1%)	11	(13.9%)	0.463
New postoperative cerebrovascular events	15	(4.8%)	8	(7.8%)	0.252	6	(7.6%)	4	(5.1%)	0.499
*Stroke*	14	(4.4%)	5	(4.9%)	0.862	6	(7.6%)	3	(3.8%)	0.294
*Intracranial bleeding*	1	(0.3%)	3	(2.9%)	0.077	0	(0%)	1	(1.3%)	0.238
Postoperative AKI	107	(34.0%)	47	(45.6%)	**0.035**	33	(41.8%)	32	(40.5%)	0.872
Re-exploration for bleeding	45	(14.3%)	26	(25.2%)	**0.009**	10	(12.7%)	17	(21.5%)	0.129
Tracheostomy	38	(12.1%)	21	(20.4%)	**0.036**	10	(12.7%)	15	(19.0%)	0.276
Time of ventilation (h)	18.2	[11–68]	32.8	[17–120]	**< 0.001**	16.8	[12–46]	28.3	[15–113]	**0.017**
ICU stay (d)	4.0	[2.0–8.0]	5.0	[3.0–13.0]	**0.009**	3.0	[1.0–6.0]	5.0	[2.0–12.0]	**0.042**
Hospital stay (d)	12.0	[8.0–16.0]	14.5	[10.0–20.0]	**0.007**	12.0	[7.0–17.0]	15.0	[11.0–20.0]	**0.015**
1-YEAR OUTCOME										
1-year mortality	53	(16.8%)	34	(33.0%)	**< 0.001**	11	(13.9%)	23	(29.1%)	**0.020**
Re-admission to hospital	94	(29.8%)	33	(32.0%)	0.478	31	(39.2%)	23	(29.1%)	0.835
Relapse of endocarditis	10	(3.2%)	3	(2.9%)	0.938	3	(3.8%)	2	(2.5%)	1.000
New pacemaker*	5	(1.6%)	4	(3.9%)	0.177	2	(2.5%)	1	(1.3%)	0.792
AKI during follow-up	23	(7.3%)	13	(12.6%)	0.071	11	(13.9%)	8	(10.1%)	0.883

Data presented as mean ± standard deviation, number (percent) or median [IQR], respectively. *AKI*, acute kidney injury; *ICU*, intensive care unit; *IQR*, interquartile range; *TIA,* transient ischemic attack; * for AV-higher grade

**Fig. 2:**
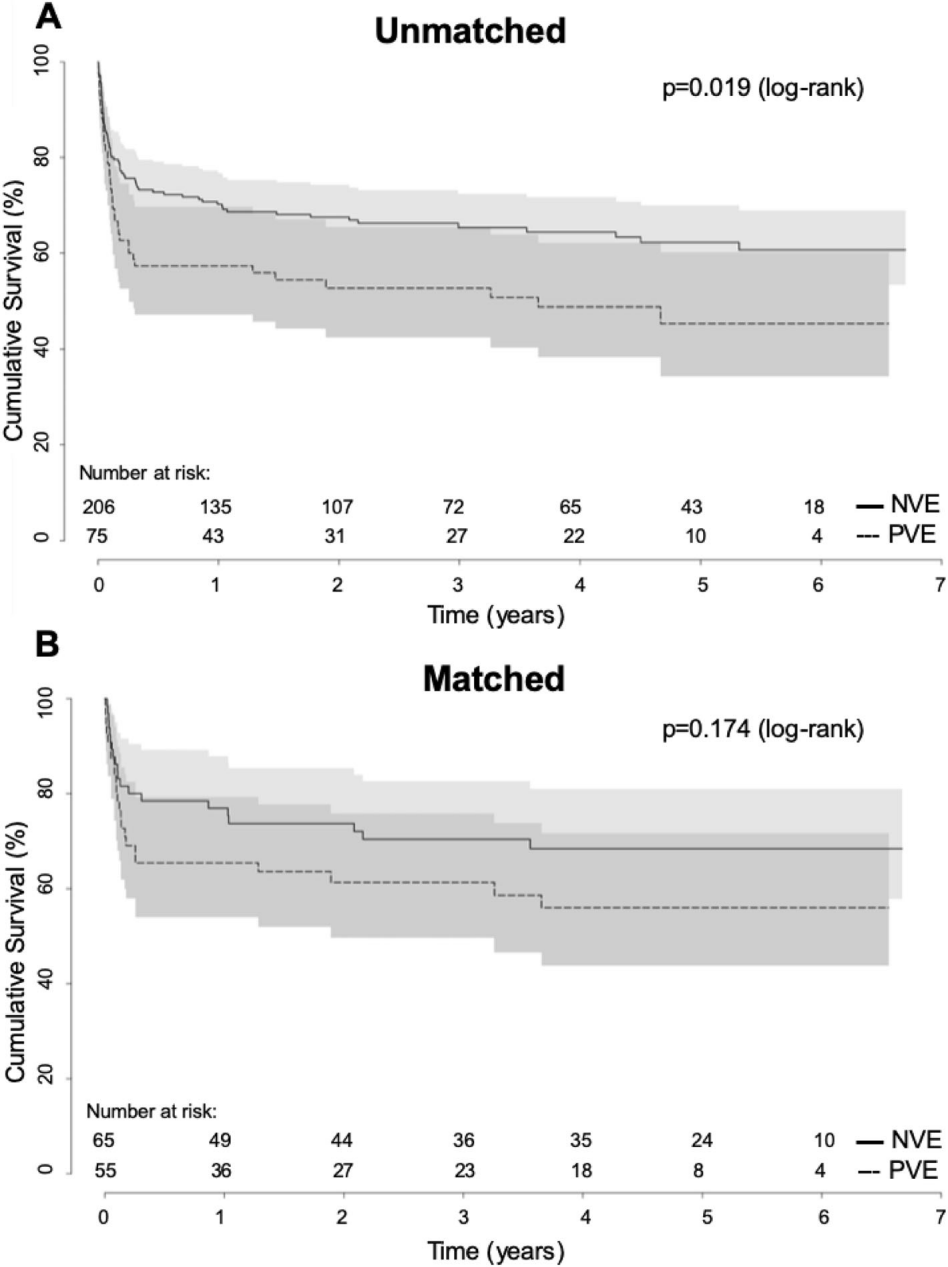
Long-term survival of patients undergoing cardiac surgery for native (NVE) versus prosthetic valve endocarditis (PVE). **a** Higher long-term mortality of patients with PVE compared to NVE. **b** After propensity-matching for relevant preoperative risk factors comparable long-term survival between NVE and PVE patients

In addition, 1-year mortality was significantly higher in PVE (29.1%) compared to NVE (13.9%, *p* = 0.020) in the matched cohort. Kaplan-Meier survival analysis revealed a significantly decreased long-term survival of patients undergoing surgery for PVE compared to NVE in the unmatched cohort (log-rank *p* = 0.019; Fig. 2a). After propensity matching no significant difference with regard to long-term survival could be found (log-rank *p* = 0.174; Fig. 2b).

### Factors associated with mortality

Univariate analysis revealed the following preoperative variables as factors associated with 30-day mortality: preoperative AKI, PVE, preoperative sepsis, perivalvular abscess, IE with *Staphylococcus aureus* (Additional file 1: Table S2).Stepwise multivariable logistic regression analysis revealed perivalvular abscess (OR 1.864 [1.002–3.465]; *p* = 0.049), preoperative AKI (OR 2.720 [1.307–5.657]; *p* = 0.007) and preoperative sepsis (OR 2.281 [1.123–4.636]; *p* = 0.023) as independent risk factors for 30-day mortality. In addition, PVE was independently predictive for 30-day mortality (OR 2.699 [1.496–4.871]; *p* = 0.001) (Table 4).

**Table 4:** Multivariable analysis of risk factors associated with 30-day mortality in patients undergoing surgery for IE

INDEPENDENT RISK FACTORS FOR 30-DAY MORTALITY			
	OR	95%CI	P value
PVE	2.699	1.496–4.871	0.001
Preoperative AKI	2.720	1.307–5.657	0.007
Preoperative sepsis	2.281	1.123–4.636	0.023
Perivalvular abscess	1.864	1.002–3.465	0.049

*CI,* confidence intervall; *IE* infective endocarditis; *OR,* odds ratio

### Patients with perivalvular abscess

Patients with perivalvular abscess showed no statistically significant differences regarding preoperative characteristics except a higher rate of peripheral vascular disease (12.2% vs. 6.2%, *p* = 0.033). Regarding the manifestation of IE, we found no difference in the distribution of underlying causative microorganisms. But patients with perivalvular abscess were diagnosed more with IE of the aortic valve (76.4% vs. 49.5%; *p* < 0.001) whereas patients without perivalvular abscess showed more mitral valve IE (56.0% vs. 33.8%; p < 0.001). Operation, CPB and crossclamp time were significantly longer in patients with perivalvular abscess due to the higher complexity of operation.

Patients with perivalvular abscess showed an impaired perioperative outcome with a significantly higher 30-day mortality (17.7% vs. 8.0%; *p* = 0.003), a higher need for postoperative pacemaker implantation for higher AV-grade (15.5% vs. 7.3%; *p* = 0.007), an increased rate of postoperative cerebrovascular events (8.8% vs. 4.0%; *p* = 0.042) and postoperative AKI (45.6% vs. 33.1%; *p* = 0.012). However, perivalvular abscess seems not to influence 1-year mortality (20.9% vs. 22.3%; *p* = 806) and postoperative long-term complications, such as readmission to hospital, relapse of IE or AKI during the follow-up (Table 5).

**Table 5:** Characteristics and outcomes of patients with and without perivalvular abscess

	- Perivalvular abscess (*n* = 275)	+ Perivalvular abscess (*n* = 148)	P value
Age	64.3 [50.6–72.9]	65.0 [50.3–74.0]	0.778
Female sex	61 (22.3%)	43 (29.1%)	0.122
BMI	25.8 [23.5–28.3]	25.4 [23.4–28.7]	0.597
BSA	1.98 [1.83–2.12]	1.96 [1.74–1.96]	0.309
COPD	26 (9.5%)	14 (9.5%)	0.999
Diabetes	78 (28.4%)	39 (26.4%)	0.659
Peripheral vascular disease	17 (6.2%)	18 (12.2%)	0.033
Preoperative AKI	156 (56.7%)	90 (60.8%)	0.417
Coronary artery disease	71 (25.8%)	46 (31.1%)	0.248
Immunosuppression	3 (1.1%)	4 (2.7%)	0.229
HIV	6 (2.2%)	4 (2.7%)	0.739
Alcohol abuse	26 (9.5%)	15 (10.1%)	0.821
Intravenous drug abuse	19 (6.9%)	9 (6.1%)	0.774
History of neoplasm	27 (9.8%)	16 (10.8%)	0.747
LVEF			
*< 30%*	4 (1.5%)	5 (3.4%)	0.201
*30–50%*	61 (22.8%)	33 (22.4%)	0.942
*> 50%*	203 (75.7%)	109 (74.1%)	0.809
MICROBIOLOGY			
Positive Blood culture	224 (89.6%)	119 (84.4%)	0.132
Streptococcus spp.	69 (25.1%)	26 (17.6%)	0.077
Staphylococcus spp.	83 (30.2%)	51 (34.5%)	0.367
Staph aureus	61 (22.2%)	33 (22.3%)	0.978
CoNS	23 (8.4%)	19 (12.8%)	0.142
Enterococcus spp.	39 (14.2%)	22 (14.9%)	0.849
ECHOCARDIOGRAPHY			
Vegetation	226 (82.5%)	110 (74.8%)	0.062
Leftsided IE			
Aortic valve	136 (49.5%)	113 (76.4%)	< 0.001
Mitral valve	154 (56.0%)	50 (33.8%)	< 0.001
Rightsided IE			
Tricuspid valve	19 (6.9%)	4 (2.7%)	0.069
Pulmonary valve	1 (0.7%)	1 (0.4%)	0.663
SYMPTOMS			
Fever	169 (61.5%)	107 (72.3%)	0.026
Sepsis	136 (49.5%)	84 (56.8%)	0.152
IE-related neurologic complications	85 (30.9%)	43 (29.1%)	0.692
Septic embolism	99 (36.1%)	47 (32.2%)	0.658
Cardiogenic shock	31 (11.3%)	25 (16.9%)	0.104
OPERATION			
Operation time (min)	195 [155–245]	230 [178–314]	< 0.001
CPB time (min)	106 [83–142]	138 [100–182]	< 0.001
Crossclamp time (min)	69 [53–95]	87 [67–115]	< 0.001
30-DAY OUTCOME			
30-day mortality	22 (8.0%)	26 (17.7%)	0.003
Myocardial infarction	1 (0.4%)	1 (0.7%)	0.663
New pacemaker *	20 (7.3%)	23 (15.5%)	0.007
New postoperative cerebrovascular events	11 (4.0%)	13 (8.8%)	0.042
Postoperative AKI	91 (33.1%)	67 (45.6%)	0.012
Re-exploration for bleeding	43 (15.7%)	28 (18.9%)	0.398
Tracheostomy	33 (12.0%)	26 (17.7%)	0.108
Time of ventilation (h)	19.8 [12.0–68.9]	26.6 [12.3–117.6]	0.118
ICU stay (d)	4.0 [2.0–8.0]	5.0 [2.0–10.0]	0.103
Hospital stay (d)	12.0 [9.0–17.0]	12.0 [9.0–17.0]	0.992
1-YEAR OUTCOME			
1-year mortality	35 (22.3%)	18 (20.9%)	0.806
Re-admission to hospital	86 (58.5%)	44 (55.0%)	0.610
Relapse of endocarditis	10 (6.9%)	4 (5.3%)	0.631
AKI during follow-up	23 (15.9%)	14 (18.2%)	0.659

Data presented as number (percent) or median [IQR], respectively. *AKI,* acute kidney injury; *BMI,* body mass index; *BSA*, body surface area; *COPD,* chronic obstructive pulmonary disease; *HIV*, human immunodeficiency virus; *ICU,* intensive care unit; *IE,* infective endocarditis; *IQR*, interquartile range; *for higher AV-grade

## Discussion

### Incidence of PVE

Although the incidence of PVE is relatively low, there is an increasing number of patients with PVE [15]. PVE occurs in 1–6% of patients with valve prostheses [4–6] and accounted for over 20% of all IE cases in a prospective, multicenter, international registry, reflecting a considerably higher proportion of PVE compared to earlier reports [4]. The proportion of PVE to NVE is comparable with our data, including 24.6% of PVE cases in our cohort of patients undergoing surgery for IE.

### Postoperative outcome of patients undergoing surgery for PVE compared to NVE

Our retrospective analysis revealed PVE as independent risk factor for mortality. PVE patients of our cohort had a significantly higher 30-day mortality compared to NVE patients (21.4% vs. 8.3%; *p* < 0.001). These findings were also reported by Romano et al. who found a higher in-hospital mortality in their PVE group, compared to their NVE group (24.2% vs. 6.6%, *p* < 0.0001) [2]. Even after propensity-matching, we still observed a 4-fold increased 30-day mortality in PVE compared to NVE.

Operation for PVE in comparison with NVE has been linked to worse outcomes and the 30-day mortality remains high. Our data demonstrate that the higher mortality of patients undergoing surgery PVE compared to NVE occurs in the early postoperative phase and up to one year postoperatively. Afterwards the Kaplan Meier curve shows parallel slopes, thus a similar long-term survival beyond the first year after surgery. This is in line with findings of Alonso-Valle et al.*,* who found highest mortality rates during the first 3 months. After this period the survival of the patients remained stable [6]. Likewise, Manne et al. found a significantly higher 30-day mortality (13% vs 5.6%, *p* < 0.01) in patients with PVE compared to NVE, but long-term survival was not significantly different (35% vs. 29%, *p* = 0.19) [16], which is similar in our study. In addition, data of the Cleveland Clinic suggest, that early mortality is significantly higher in patients with PVE, whereas 1-year survival is comparable [16]. This is in line with the findings of Edlin et al., reporting on similar long-term survival in patients with PVE compared to NVE [15].

Several factors have been associated with the increased mortality in PVE including increased age [17], female sex [18], congestive heart failure [19], staphylococcal infection [4] and renal dysfunction [20]. In several other previous studies, periannular complications were also associated with significantly higher in-hospital mortality [1, 4, 6, 18]. In line with this, our multivariate analysis revealed PVE, preoperative AKI, preoperative sepsis and perivalvular abscess as independent risk factors for mortality.

### Perivalvular infection

In our cohort we could detect significantly more perivalvular abscesses in 60.2% of PVE compared to 27.0% of NVE patients (*p* < 0.001). This is in line with other studies reporting on periannular extensions in 56–100% of patients with PVE [10]. Due to frequent presence of perivalvular infection, surgery for PVE is technically more demanding than for NVE, as it requires radical debridement and complex surgical intervention [8, 10]. This is reflected in the longer CPB and crossclamp times in our PVE group, which might be a surrogate parameter for the complexity of procedure. In several previous studies, periannular complications were associated with significantly higher in-hospital mortality [1, 4, 6, 18, 21]. In accordance, we found a 30-day mortality rate of 17.7% in patients with perivalvular abscess, which was significantly higher compared to patients without perivalvular abscess (8.0%) (*p* = 0.003). Besides the higher 30-day mortality, patients with perivalvular abscess suffered from more postoperative complications, such as the need of postoperative pacemaker implantation, new postoperative cerebrovascular events and postoperative AKI. Our data suggest, that perivalvular abscess influences 30-day mortality and perioperative complications, but has no relevant impact on 1-year mortality or long-term complications in terms of readmission to hospital, relapse of IE or AKI during the follow-up. Therefore, we suppose that the difference in survival during the first year after surgery for PVE and NVE might be attributed to the higher prevalence of perivalvular abscess in PVE patients, which is associated with a higher rate of 30-day mortality and perioperative complications, but has no impact on 1-year mortality, readmission rate or relapse of IE.

### Impaired timely diagnosis of PVE

Timely diagnosis of PVE can be difficult as clinical presentation is frequently atypical and results of blood cultures and echocardiography are more often negative, leading to lower sensitivity of the Duke criteria [7–9]. This is supported by our data, showing a significantly lower rate of vegetations in echocardiography for patients with PVE (70.9% vs. 82.2%; *p* = 0.017). Hence, we suggest that early detection and diagnosis of PVE is essential to prevent an increase of perivalvular destruction. Several studies have investigated the sensitivity and specificity of PET/CT or SPECT/CT imaging in patients with suspected IE [22, 23]. PET-CT increased the sensitivity and specificity of the modified Duke criteria from 70 to 95%, reducing the number of patients with „possible IE “from 56 to 32% [22, 23]. A combination of TTE, TEE and CT can increase the sensitivity for the detection of valvular and perivalvular complications in IE [11]. Therefore, early detection of perivalvular lesions by using CT imaging should be considered in suspected IE as implemented in the recent ESC guidelines for the management of IE [9].

### Distribution of causative microorganisms

Besides more perivalvular infection, the spectrum of microorganisms that cause PVE seems to differ from those detected in NVE. Several studies reported, that *Staphylococci* were more frequent in PVE, with a predominance of *Staphylococcus aureus* or *CoNS* [4, 24, 25]. Previous studies revealed, that IE due to *Staphylococcus aureus* is characterized by aggressive disease with increased risk of embolism, stroke and mortality [26]. However, the incidence of *Staphylococcus aureus* IE was comparable between PVE and NVE patients in our cohort. Nevertheless, we included infection with *Staphylococcus aureus* as matching variable in our propensity matching*.* Although we could not find a difference in the incidence of *Staphylococcus aureus* IE, patients with PVE were diagnosed more often with *CoNS* compared to NVE patients. As ubiquitous skin commensals, CoNS are often associated with health-care contact and invasive procedures [7]. They colonise indwelling lines and devices and are the most common isolate in early prosthetic valve endocarditis [6, 7, 25]. Staphylococci and gram-negative microorganisms have been suspected to occur as nosocomial perioperative infection during a period when the prosthetic valve is not completely endothelialized [5]. Therefore strict hygiene during health-care contact and invasive procedures in patients with prosthetic valves is essential.

### Limitations

Our study has several limitations that need to be considered for the interpretation of the results. First, the retrospective design and analysis of a limited number of patients from a single center institution reduces the generalizability. In addition, a powerful multivariable analysis of all possible predictors was restricted by the total patient number. Moreover, although we attempted to correct for confounders between groups by propensity matching, there might still remain differences between groups. Finally, we included a very recent patients’ cohort, therefore the 1-year follow-up is not completed in all patients. Nevertheless, despite the limited sample size, our study still comprises a relatively large IE cohort compared to the recent literature and provides meaningful results due to propensity matching.

## Conclusions

Considering patients with similar risk profiles, our analysis revealed PVE as an independent risk factor for mortality. PVE was associated with significantly higher 30-day and 1-year mortality compared to NVE. After propensity-matching 30-day mortality was still 4-fold increased in PVE compared to NVE.

The higher incidence of perivalvular abscess seems to determine clinical outcome. Patients with perivalvular abscess showed a significantly higher 30-day mortality and perioperative complications, whereas perivalvular abscess seems to have no relevant impact on 1-year mortality, the rate of readmission or relapse of IE. We suggest, that the higher prevalence of perivalvular abscess in patients undergoing surgery for PVE might have an important impact on the impaired outcome during the first year after surgery for PVE. Therefore, early detection and diagnosis of patients with PVE is crucial and CT imaging should be evaluated in suspected PVE.

## Supplementary information

**Additional file 1: Table S1** Perioperative Characteristics. **Table S2** Univariate analysis of preoperative risk factors associated with 30-day mortality in patients undergoing surgery for IE

## Data Availability

All data generated and/or analysed during this study are included in this published article and its supplementary files.

## Abbreviations

AKI: Acute kidney injury
CI: Confidence interval
CoNS: Coagulase-negative staphylococci
CPB: Cardiopulmonary bypass
ESC: European society of cardiology
IE: Infective endocarditis
IQR: Interquartile range
NVE: Native valve endocarditis
OR: Odds ratio
PVE: Prosthetic valve endocarditis
SD: Standard deviation
