# Supplementary material for: Higher incidence of perivalvular abscess determines perioperative clinical outcome in patients undergoing surgery for prosthetic valve endocarditis
Source: BMC Cardiovasc Disord. 2020 Feb 3;20:47. doi: 10.1186/s12872-020-01338-y (PMC6998844; doi:10.1186/s12872-020-01338-y)
Supplement: Supplementary file 1 — Additional file 1: Table S1 Perioperative Characteristics. Table S2 Univariate analysis of preoperative risk factors associated with 30-day mortality in patients undergoing surgery for IE [file 12872_2020_1338_MOESM1_ESM.docx]

| **SUPPL. TABLE 1. Perioperative Characteristics** | | | | | | | | | | |
| --- | --- | --- | --- | --- | --- | --- | --- | --- | --- | --- |
|  | **Entire cohort** | | | | | **Propensity matched cohort** | | | | |
|  | **NVE**  **(n = 315)** | | **PVE**  **(n = 103)** | | **P value** | **NVE**  **(n= 79)** | | **PVE**  **(n= 79)** | | **P value** |
| **TIMING OF OPERATION** |  |  |  |  |  |  |  |  |  |  |
| Emergent (< 24h) | 24 | (7.6%) | 6 | (5.8%) | 0.540 | 2 | (2.5%) | 4 | (5.1%) | 0.405 |
| Urgent (24-72h) | 35 | (11.1%) | 10 | (9.7%) | 0.690 | 10 | (12.7%) | 9 | (11.4%) | 0.807 |
|  |  |  |  |  |  |  |  |  |  |  |
| **OPERATION** |  |  |  |  |  |  |  |  |  |  |
| Operation time (min) | 185 | [149-235] | 274 | [226-326] | **<0.001** | 181 | [145-222] | 275 | [229-325] | **<0.001** |
| CPB time (min) | 104 | [80-139] | 159 | [126-204] | **<0.001** | 97 | [76-130] | 166 | [127-204] | **<0.001** |
| Crossclamp time (min) | 70 | [53-95] | 95 | [70-125] | **<0.001** | 68 | [55-85] | 95 | [71-125] | **<0.001** |

Data presented as mean ± standard deviation, number (percent) or median [IQR], respectively. *CPB,* cardiopulmonary bypass

| **SUPPL. TABLE 2. Univariate analysis of preoperative risk factors associated with 30-day mortality in patients undergoing surgery for IE** | | | |  |
| --- | --- | --- | --- | --- |
|  | **Entire cohort** | | | |
|  | **OR** | **95%CI** | **P value** | |
| Female gender | 1.869 | 0.984-3.551 | 0.053 | |
| Age >65 years | 1.596 | 1.058-2.408 | **0.010** | |
| Diabetes | 1.229 | 0.640-2.357 | 0.535 | |
| Peripheral vascular disease | 2.194 | 0.899-5.354 | 0.105 | |
| Preoperative AKI | 3.066 | 1.484-6.335 | **0.002** | |
| Intravenous drug abuse | 0.582 | 0.134-2.533 | 0.439 | |
| Neurologic symptoms | 0.751 | 0.377-1.496 | 0.414 | |
| PVE | 3.009 | 1.620-5.587 | **<0.001** | |
| Previous pacemaker implantation | 2.507 | 0.953-6.593 | 0.083 | |
| Preoperative sepsis | 2.481 | 1.289-4.774 | **0.005** | |
| Vegetation | 2.294 | 0.878-5.988 | 0.082 | |
| Perivalvular abscess | 2.471 | 1.346-4.537 | **0.003** | |
| Perforation | 1.281 | 0.658-2.492 | 0.466 | |
| Fistula | 2.200 | 0.591-8.183 | 0.273 | |
| IE with Staph. aureus | 1.993 | 1.039-3.823 | **0.035** | |

*CI,* confidence intervall; *IE* infective endocarditis; *OR,* odds ratio
